# Inconsistencies in the Nutrition Management of Glutaric Aciduria Type 1: An International Survey

**DOI:** 10.3390/nu12103162

**Published:** 2020-10-16

**Authors:** Laurie Bernstein, Curtis R. Coughlin, Morgan Drumm, Steven Yannicelli, Fran Rohr

**Affiliations:** 1 Department of Pediatrics, Section of Medical Genetics, University of Colorado Anschutz Medical Campus, Aurora, CO 80045, USA; curtis.coughlin@childrenscolorado.org (C.R.C.); morgan.drumm@childrenscolorado.org (M.D.); 2 Nutricia North America, Rockville, MD 20850, USA; steven.yannicelli@nutricia.com; 3 Met Ed Co, Boulder, CO 80302, USA; fran.rohr@met-ed.net

**Keywords:** glutaric aciduria type 1, glutaric acidemia type 1, nutrition, diet, lysine-restricted, protein

## Abstract

Glutaric aciduria type 1 (GA-1) is a cerebral organic aciduria characterized by striatal injury and progressive movement disorder. Nutrition management shifted from a general restriction of intact protein to targeted restriction of lysine and tryptophan. Recent guidelines advocate for a low-lysine diet using lysine-free, tryptophan-reduced medical foods. GA-1 guideline recommendations for dietary management of patients over the age of six are unclear, ranging from avoiding excessive intake of intact protein to counting milligrams of lysine intake. A 22–question survey on the nutrition management of GA-1 was developed with the goal of understanding approaches to diet management for patients identified by newborn screening under age six years compared to management after diet liberalization, as well as to gain insight into how clinicians define diet liberalization. Seventy-six responses (25% of possible responses) to the survey were received. Nutrition management with GA-1 is divergent among surveyed clinicians. There was congruency among survey responses to the guidelines, but there is still uncertainty about how to counsel patients on diet optimization and when diet liberalization should occur. Ongoing clinical research and better understanding of the natural history of this disease will help establish stronger recommendations from which clinicians can best counsel families.

## 1. Introduction

Glutaric aciduria type 1 (GA-1) is a cerebral organic aciduria characterized by a striatal injury and a progressive movement disorder [1]. The neurologic injury is often precipitated by an intercurrent illness within the first three years of life, although approximately 10–20% of affected patients have an insidious presentation [2,3,4]. Historically, 80–90% of undiagnosed and untreated patients developed striatal necrosis, although newborn screening has dramatically changed the natural history of this disease [5,6]. With early diagnosis and aggressive medical intervention during acute illness, most patients remain healthy into adulthood, emphasizing the importance of treatment.

GA-1 results from the deficiency of glutaryl-CoA dehydrogenase (GCDH, E.C. 1.3.8.6), which is a key enzyme in lysine oxidation and decarboxylation. GCDH catalyzes the oxidative decarboxylation of glutaryl-CoA to crotonyl-CoA, and the deficiency of GCDH results in the accumulation of glutaryl-CoA, glutaconic acid, glutaric acid and 3-hydroxyglutaric acid [7]. Although the pathophysiology of this disorder is still incompletely understood, the accumulation of 3-hydroxyglutaric acid is thought to play a role in cell damage and development of striatal injury [8,9,10]. Current therapeutic strategies focus on limiting the accumulation of metabolites through aggressive management of illness to limit catabolism and the use of carnitine to enhance detoxification of glutaryl-CoA and prevent a secondary carnitine deficiency [5,11,12]. Similarly, dietary-based treatments reduce precursor amino acids lysine and tryptophan and subsequently decrease the accumulation of glutaric acid and 3-hydroxyglutaric acid. Patients with GA-1 have been managed with intact protein-restricted diets for over forty years, although it is often unclear how clinicians implement these chronic dietary-based therapies.

Initially, patients who presented to care after an acute neurologic injury were treated with generalized protein restriction, which resulted in mild improvement in biochemical parameters and limited improvement in clinical symptoms [1,13,14]. In small case series, presymptomatic protein restriction was associated with a decreased risk of neurologic injury, although these results were confounded by concomitant treatment with acute illness management and carnitine supplementation [3,5]. Furthermore, patients who were treated with only acute management and carnitine also remained asymptomatic [3,15], which raised questions about the impact of protein restriction on the development of neurologic disease [16]. Despite these concerns, dietary-based therapies remain central to the treatment of patients with GA-1 [11,17,18].

Over time, nutrition management shifted from a general restriction of intact protein to the targeted restriction of lysine and tryptophan [19,20]. The goal of both therapeutic strategies is to reduce lysine intake while ensuring adequate intakes of other essential and nonessential amino acids, vitamins and minerals [19]. Recent guidelines advocate for a low-lysine diet through the use of lysine-free, tryptophan-reduced amino acid supplements (medical foods) [11], and it is unclear how many patients are currently managed with a generalized intact protein restriction [21].

Adults with GA-1 can present with a variety of neurologic symptoms, including chronic headaches, peripheral neuropathy, white matter abnormalities and subependymal nodules [22,23,24,25,26]. These late-onset presentations are consistent with a chronic neurologic deterioration as opposed to the acute encephalopathic crisis and subsequent striatal injury that occurs in the first few years of life. As a result, many clinicians have liberalized patient’s intact protein restriction over time, although there are few recommendations on how and when to change a patient’s treatment. Guidelines for dietary management of patients over the age of six are unclear and range from avoiding excessive intake of intact protein [17] to recommendations for controlling protein intake with a focus on protein sources with low lysine content [11].

Observational studies have demonstrated the importance of aggressive management, especially in an asymptomatic infant [6,27,28], although the implementation of dietary recommendations is often not clear. Herein we present results from an international empiric study on the nutritional management of patients with GA-1.

## 2. Materials and Methods

A survey on the nutrition management of GA-1 was developed with the goal of understanding approaches to diet management for patients identified by newborn screening under age 6 years compared to management after diet liberalization, as well as to gain greater insight into how clinicians define diet liberalization. The 22-question survey was created by the authors and reviewed by several experts in GA-1 (Supplementary Table S1). The survey was sent to approximately 300 health care professionals enrolled in a metabolic dietitian listserv (PNO-METAB-L@LISTSERV.CC.EMORY.EDU). Participants were asked to respond only if they currently manage patients with GA-1.

## 3. Results

Seventy-six dietitians (25% of possible responses) responded to the survey. Of these, 58 respondents (76%) practice in the US and 18 (24%) practice outside of the US (Argentina—5, Canada 4, Australia—2, Saudi Arabia—3, Ukraine—1). Eighty-six percent of respondents manage patients with GA-1 who were identified by newborn screening, 61% manage patients who were identified after a striatal injury, and 39% manage patients with late-onset GA-1.

### 3.1. Management of Patients with GA-1 Who Are under 6 Years of Age and Were Identified by Newborn Screening

#### 3.1.1. Breastfeeding

The majority of clinicians surveyed allow infants with GA-1 to receive a limited amount of breastmilk in their diets; 50% of respondents allow feeding at the breast, and 20% allow expressed breastmilk to be offered from the bottle. Ten percent do not recommend breastfeeding. Another 20% of respondents chose “other”; many commented that they had not yet faced the decision about breastfeeding, either because they have not yet had the opportunity or because their patients were already formula fed when they presented.

#### 3.1.2. Medical Food

Medical foods (also referred to as *foods for special medical purposes* or protein substitutes) are formulations containing amino acids (excluding lysine, low tryptophan) as the source of nitrogen, as well as calories, vitamins and minerals and are designed for nutrition management of patients with GA-1. Medical foods may also be supplemented with L-arginine. Survey results for patients under six years of age showed that 87% of respondents recommend the use of medical foods for patients with GA-1; 13% do not. Of those who do not use medical food, 63% responded that they counsel their patients to restrict intact protein, and 37% do not restrict.

#### 3.1.3. Counting Intact Protein

For patients who are instructed to count intact protein intakes, most (76%) count grams of protein from food, whereas only 20% count milligrams of lysine, and 6% use an “exchange” system. Many clinicians commented that their approach is patient-dependent and that they may start with counting lysine and then move to counting protein as the child ages.

#### 3.1.4. Arginine Supplementation

Most clinicians (95%) do not recommend L-arginine supplementation (other than what is contained in medical food).

### 3.2. Diet Liberalization

#### 3.2.1. Definition of Diet Liberalization

**Figure 1:**
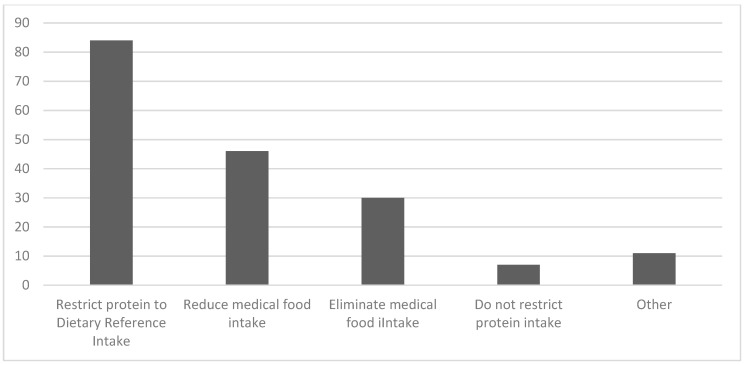
Survey responses to defining diet liberalization in glutaric aciduria type 1 (GA-1) (% of responses in each category).

As seen in Figure 1, diet liberalization in GA-1 has a wide range of meanings to clinicians. Typically (for 84% of respondents), diet liberalization means “some restriction of food protein to provide the US dietary reference intake (DRI) or similar standard recommendation for protein intake if outside the US)”. Diet liberalization also entails the reduction of medical food (46% of respondents) or its elimination (30% of respondents). For 7% of respondents, liberalization means no restriction of food protein.

#### 3.2.2. Age of Diet Liberalization

**Figure 2:**
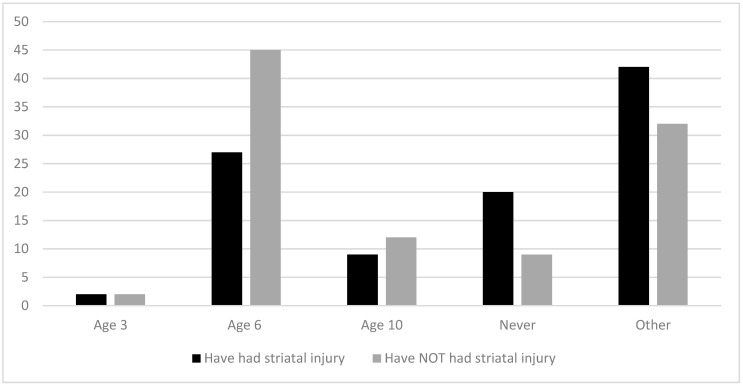
Recommended age of diet liberalization for patients with GA-1 injury (% of survey responses for each age category).

The question “When does your clinic liberalize the diet?” was asked for both patients who have had an acute encephalopathic crisis resulting in striatal injury and those who have not. As seen in Figure 2, the most common answer for both groups was “after age 6 years”, with 27% of respondents recommending diet liberalization for those with striatal injury at this age. Forty-five percent of respondents recommend diet liberalization after age 6 years for patients who have not had striatal injury. Responses ranged from recommending diet liberalization as early as “after age 3 years” (2% of respondents in both groups) to never (20% for those with striatal injury and 9% for those patients who have not had a striatal injury).

#### 3.2.3. Definition of a Protein-Controlled Diet

Eighty-five percent of respondents consider “protein-controlled diet” to mean providing only the amount of protein recommended by the US DRI (or similar standard recommendation for protein intake if outside the US). For 44% of respondents, “protein-controlled diet” also meant that the clinician prescribes a certain number of grams of protein that the patient counts and 27% consider “protein- controlled diet” to mean not allowing meat or high biological value protein.

#### 3.2.4. Tracking Protein Intake after Diet Liberalization

Once the diet has been liberalized, most clinicians (63%) counsel patients to count grams of protein, 20% suggest that patients count “servings” of higher protein foods, and 10% do not have patients count protein at all (full liberalization). The remaining 7% replied “Other”, and comments indicated that the approach depends on the patient.

#### 3.2.5. Goals for Plasma Lysine Concentrations

**Figure 3:**
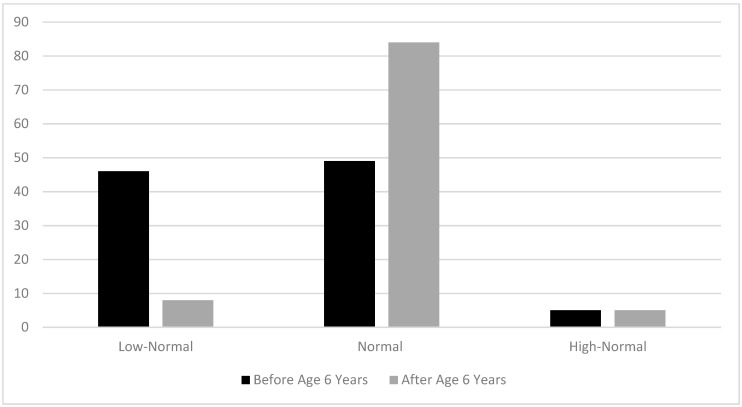
Goals for plasma lysine concentrations in patients with GA-1 before and after age 6 years (% of survey responses for each category).

Figure 3 shows the goals for plasma lysine concentrations that clinicians aim to achieve in patients before and after age 6 years. For patients before age 6 years, almost half of the respondents (46%) stated that they aim to keep plasma lysine in the low normal range; after age 6 years only 5% do so, and the vast majority of respondents (84%) aim for normal lysine concentrations.

#### 3.2.6. Diet Monitoring

Both before and after diet liberalization, in order to determine the need for a change in diet, most respondents monitor plasma amino acids (93% and 92%, respectively) and anthropometrics (91% and 88%, respectively). Very few respondents (9% before liberalization and 12% after liberalization) monitor plasma 3-hydroxy glutaric acid. Likewise, neurocognitive status is monitored by 23% of respondents following patients before the diet is liberalized, and by 34% of respondents after the diet has been liberalized.

## 4. Discussion

For patients identified with GA-1 by newborn screening, striatal injury can be prevented by appropriate management, including aggressive intervention during illness in early life [5,27,29,30]. When coupled with medical and nutrition management, newborn screening for GA-1 can result in positive clinical outcomes [30]. Treatment guidelines during illness are well accepted; however, chronic approaches to nutrition management are not consistent among institutions.

Guidelines for GA-1 management have been published and revised [11,17,18]. Approaches to management are disparate based on established clinical dogma and experience. This is not uncommon in rare metabolic disorders due to the dearth of the full understanding of the natural history of the disease. In the current survey, the authors focused on nutritional management approaches to identify current real-world experiences and how they compare to established guidelines. The survey suggests that inconsistencies in practice exist in several major areas, including what diet liberalization means, when the diet should be liberalized and nutrition counseling for patients both on and off diet.

To most of the clinicians surveyed, diet liberalization and a “protein-controlled diet” means restricting intact protein intake to the standard reference, defined in this survey as the US Dietary Reference Intake [31], or an equivalent international reference. Yet, to many, it also means reducing the intake of medical food (as is likely to occur as intact protein increases), and to some, liberalization means no restriction of protein.

The DRI for protein intake for an adult is 56 g of protein per day. However, US diet intake data show that for most, meeting but not exceeding the DRI would mean consuming far less protein than the typical adult. National Health and Nutrition Examination Survey (NHANES) data show that protein intake was 82.3 ± 0.8 g/day (mean ± SE), almost half of which is consumed as animal protein [32]. Moreover, for children, the gap between the minimum amount of protein required and typical intakes is even greater. NHANES results indicate that for children 4–8 years of age, the average daily protein intake was 61.6 + 0.5 g (mean ± SE), yet the DRI recommended dietary allowance for this age group is 19 g of protein [33]. The average protein intake was 13.6% of energy intake, which is within the acceptable recommended macronutrient distribution for protein of 10–35% of energy intake and, therefore, does not represent excessive protein intake [33]. Thus, if the recommendation is for a patient with GA-1 to liberalize diet at age six years and limit protein intake to the DRI, the patient’s protein intake would be very low compared to average intakes, not considered to be a free diet. The need for individualization of nutrition management, including assessing sufficient protein adequacy, is in line with Boy et al. [11].

The survey results showing that 87% of respondents use amino acid-based medical foods as an integral part of diet plan in children under 6 years of age is complementary to the current and previous guidelines [11,18]. Positive growth and neurological outcomes have been described when instituting lysine-free amino acid medical foods in conjunction with low lysine foods, carnitine and emergency treatment [30,34]. Researchers have reported muted clinical effects when quantifying intact proteins alone compared to lysine in the absence of medical foods [5,35].

Counting intact protein (grams) is simpler than quantifying lysine (milligrams), but it is less precise and does not consider the quality of protein consumed nor differences in lysine content of common foods. Despite these concerns, conversion to a food protein-counting method is often used in nutrition management as patients age and is in line with current guidelines [11].

The overwhelming majority of survey respondents do not recommend supplementing the diets of patients with L-arginine per se, but rather calculate the contribution from the amino acid-based medical foods. The amount of arginine from medical foods varies, as does arginine from protein-containing foods depending on the type and amount of protein consumed. Consequently, L-arginine intake can have significant variance that must be calculated by the clinician. Despite the trend to reduce and even eliminate amino acid-based medical foods, the main contributor of arginine, guidelines do not recommend supplementing arginine [11,18].

The revised guidelines strongly recommend a low lysine diet with amino acid-based medical foods up to age six years [11]. After age six years, the recommendation is to transition to an individualized age-appropriate “protein-controlled” diet. Despite this recommendation, fewer than half of survey respondents (45%) recommend diet liberalization after age 6 years in patients who were identified via newborn screening and have not had a striatal injury. Twenty percent of respondents “never” liberalize diet. The time of liberalization is variable and individualized. Some respondents approach liberalization slowly at six years or older, and they may continue the use of medical food. One reason for the hesitation in liberalizing the diet may be the concern among families and patients who are reluctant to change nutrition management due to fear of neurodegeneration, even if the patient has had striatal injury.

This survey has the limitations that respondents were self-selected and may not be representative of metabolic clinicians; in particular, there was only one respondent from Europe. Survey responses about nutrition management practices were not linked to outcomes in patients with GA-1.

## 5. Conclusions

GA-1 is challenging to manage nutritionally because the typical biomarkers, such as amino acids or organic acids, are not good correlates for assessing the appropriateness of a patient’s diet, nor are they related to patient outcomes. Consequently, nutrition management approaches to patients with GA-1 are divergent among clinicians. Although in this survey, there was agreement among responders to the current GA-1 guidelines, there is still uncertainty of how to best counsel families and patients on diet optimization and time for liberalization. What is needed is a clearer definition of what “liberalized”, and “protein-controlled” diets mean as well as consideration as to whether only meeting minimum protein requirements is optimal nutrition management once the diet is liberalized. Ongoing clinical research, contributing to the natural history of this disease, will help establish stronger recommendations from which clinicians can best counsel families.

## Supplementary Materials

The following are available online at https://www.mdpi.com/2072-6643/12/10/3162/s1, Table S1: Glutaric Aciduria Type 1 (GA-1) Questionnaire.
