# Supplementary material for: Inconsistencies in the Nutrition Management of Glutaric Aciduria Type 1: An International Survey
Source: Nutrients. 2020 Oct 16;12(10):3162. doi: 10.3390/nu12103162 (PMC7602866; doi:10.3390/nu12103162)
Supplement: Supplementary file 1 [file nutrients-12-03162-s001.pdf]

Supplementary Table S1 Glutaric Aciduria Type 1 (GA-1) Questionnaire.

Section 1- The first set of questions are about your clinic and patient population

1. Is your clinic in the United States?
   a. Yes
   b. No
   c. If No, in which country are you located?

2. How many patients do you follow with GA-1, and how were they identified? (respond to all that apply, and include # of patients for each category)
   Total: _____
   Identified by newborn screening: ____
   Identified clinically after striatal injury: ____
   Late-onset: ____
   Identified by family studies: ____
   Other (please specify): ____

Section 2- The following questions apply to your patients with GA-1 under the age of 6 years who were identified by newborn screening

3. Do you allow breastfeeding in infants with GA-1?
   a. Yes, a limited amount of breast milk from the breast is allowed
   b. Yes, a limited amount of expressed breast milk fed from the bottle is allowed
   c. No
   d. Other (please specify)

4. For patients under 6 years of age identified by newborn screening, do you use a medical food (Food for Special Medical Purposes/Protein Substitutes) designed for managing patients with GA-1?
   a. Yes
   b. No

5. For patients under 6 years of age identified by newborn screening, do you counsel your patients who are not receiving medical food to restrict protein?
   a. Yes
   b. No
   c. Other (please specify)

6. For patients under 6 years of age identified by newborn screening, how do you counsel your patients to count protein?

a. Count lysine (mg) intake from food
b. Count protein (g) intake from food
c. Counting protein using the exchange system
d. Other (please specify)

7. For patients under 6 years of age identified by newborn screening, do you supplement your patients' diet with L-arginine (in addition to what is in the medical food)?
   a. Yes
   b. No
   c. If yes, how much do you prescribe?

8. For patients under 6 years of age identified by newborn screening, if you supplement with L-arginine when do you recommend patients take the arginine?
   a. Timed with lysine or protein intake
   b. Evenly divided throughout the day
   c. Whenever they chose to
   d. Other (please specify)

9. For patients under 6 years of age identified by newborn screening, what do you monitor to determine if the diet is appropriate? (check all that apply)
   a. Plasma Amino Acids
   b. Plasma lysine only
   c. Neurocognitive status (formal assessment)
   d. Anthropometrics
   e. Brain MRI
   f. Plasma 3-OH glutaric acid
   g. Other (please specify)

10. For patients under 6 years of age identified by newborn screening, do you aim for a certain ratio of lysine to arginine in plasma?
    a. Yes
    b. No
    c. If yes, what ratio?

11. For patients under 6 years of age identified by newborn screening, where do you aim to keep your patients' plasma lysine levels?
    a. Normal range
    b. High normal range
    c. Low normal range
    d. Other (please specify)

12. For patients under 6 years of age identified by newborn screening, what supplements do you usually recommend? (check all that apply)
    a. Multivitamin/mineral
    b. Calcium
    c. Vitamin D
    d. None
    e. Other (please specify)

<u>Section 3- The following questions address liberalization of the diet for patients with GA-1 who were identified by newborn screening</u>

13. How does your clinic define liberalizing the diet? (check all that apply)
    a. Eliminate medical food
    b. Reduce intake of medical food
    c. No restriction of food protein
    d. Some restriction of food protein to provide the Dietary Reference Intake (DRI) (or similar standard recommendation for protein if outside the US)
    e. Other (please specify)

14. When does your clinic liberalize the diet in patients with GA-1 who have been identified by newborn screening and who *have had* an acute encephalopathic crisis resulting in striatal injury?
    a. After age 3 years
    b. After age 6 years
    c. After age 10 years
    d. Never
    e. Other (please specify)

15. When does your clinic liberalize the diet in patients with GA-1 who have been identified by newborn screening and who *have not* had acute encephalopathic crisis resulting in striatal injury?
    a. After age 3 years
    b. After age 6 years
    c. After age 10 years
    d. Never
    e. Other (please specify)

16. The GA-1 recommendations for treatment (Boy et al. JIMD 2017) suggest "After age 6 years, dietary treatment should follow an age-adapted, protein-controlled protocol based on safe levels for protein intake." How would you describe a protein-controlled diet? (Check all that apply)

a. Provide only the Dietary Reference Intake for protein (or similar standard recommendation for protein if outside the US)
b. Prescribe grams of protein from food that the patient counts
c. Do not allow meat or high biological value protein
d. None of the above
e. Other (please specify)

17. Once the diet has been liberalized, how does the patient track the amount of protein consumed?
    a. Counts grams of protein
    b. Counts milligrams of lysine
    c. Counts "servings" of higher protein foods
    d. Does not count protein/full liberalization
    e. Other (please specify)

18. Once the diet has been liberalized, what do you monitor to determine if the diet is appropriate for patients whose diets have been liberalized? (check all that apply)
    a. Plasma Amino Acids
    b. Plasma lysine only
    c. Neurocognitive status (formal assessment)
    d. Anthropometrics
    e. Brain MRI
    f. Plasma 3-OH glutaric acid
    g. Other (please specify)

19. Once the diet has been liberalized, do you aim for a certain ratio of lysine to arginine in plasma?
    a. Yes
    b. No
    c. If yes, what ratio?

20. Once the diet has been liberalized, where do you aim to keep your patients' plasma lysine levels?
    a. Normal range
    b. High normal range
    c. Low normal range
    d. Other (please specify)

21. Once the diet has been liberalized, what supplements do you usually prescribe? (check all that apply)
    a. Multivitamin
    b. Calcium
    c. Vitamin D

d. None
e. Other (please specify)
